# Prevalence of DNA Mismatch Repair Deficiencies in Multiple Solid Tumor Types in China

**DOI:** 10.1111/jebm.70081

**Published:** 2025-11-28

**Authors:** Xiaohua Shi, Xianghong Yang, Jingping Yun, Xiangshan Fan, Yingyong Hou, Zhe Wang, Peng Li, Jieyu Chen, Dongxian Jiang, Longyun Chen, Yan Wang, Rubentiran Ramar, Michael Thomas Wong, Song Ling Poon, Zhiyong Liang

**Affiliations:** ^1^ Department of Pathology, Peking Union Medical College Hospital Chinese Academy of Medical Sciences and Peking Union Medical College Beijing China; ^2^ Department of Pathology Shengjing Hospital of China Medical University Shenyang Liaoning China; ^3^ Department of Pathology Sun Yat‐sen University Cancer Center Guangzhou Guangdong China; ^4^ Department of Pathology The Affiliated Drum Tower Hospital of Nanjing University Medical School Nanjing Jiangsu China; ^5^ Department of Pathology, Zhongshan Hospital Fudan University Shanghai China; ^6^ Value & Implementation, Global Medical & Scientific Affairs MSD China Shanghai China; ^7^ Precision Medicine MSD International GmbH Singapore Singapore

**Keywords:** China, DNA mismatch repair deficiency, epidemiology, immune checkpoint inhibitor, microsatellite instability, prevalence

## Abstract

**Aim:**

Microsatellite instability (MSI) as a result of deficient deoxyribonucleic acid (DNA) mismatch repair (dMMR) is a key contributor to the development of tumors with a high mutation rate and cancer‐specific neoantigens. dMMR identification can be beneficial for selection of immune checkpoint inhibitor (ICI) therapy‐eligible patients. While multiple studies have focused on dMMR prevalence in colorectal cancer (CRC), fewer investigate the prevalence of dMMR in tumor types besides CRC, especially in Chinese patients. In this study, we aimed to determine the prevalence of dMMR in China across five gastrointestinal and gynecological tumor types.

**Methods:**

Tissue samples from Chinese patients with advanced endometrial, ovarian, cervical, biliary tract, or gastric metastatic or unresectable solid tumors were tested for dMMR status using immunohistochemistry with the Ventana MMR RxDx panel. Data were analyzed to determine the prevalence of dMMR for each tumor type.

**Results:**

A total of 748 patients were included in the study, representing five tumor types. Prevalence of dMMR varied across tumor types, with an overall prevalence of 9.4%. Patients with endometrial tumors had the highest proportion of patients with dMMR at 49/164 (29.9%). Patients with cervical tumors had the lowest prevalence of dMMR with 6/221 (2.7%) patients. The prevalence of dMMR was similar across most demographic characteristics. In the dMMR population, co‐occurring MLH1 and PMS2 protein loss across all tumor types was observed most commonly, in 48/70 (68.6%) patients.

**Conclusions:**

These data highlight the importance of dMMR testing in patients with advanced solid tumors in China to optimize biomarker testing and treatment decisions.

## 1 Introduction

Deoxyribonucleic acid (DNA) mismatch repair (MMR) is a cellular process that corrects errors in DNA replication to maintain genomic integrity. The MMR pathway starts with the recognition of mismatched bases that arise during DNA replication or recombination. MMR maintains genomic stability by correcting base‐base mismatches and insertion/deletion errors in DNA [1]. Loss of MMR function can lead to genomic instability of repetitive DNA sequences known as microsatellites, leading to microsatellite instability (MSI) [2, 3]. MSI as a result of deficiency in DNA MMR (dMMR) is a key factor in the development of tumors with a high mutation rate and cancer‐specific neoantigens [4].

Identification of dMMR/high MSI (MSI‐H) can be beneficial for the selection of patients who are eligible for immune checkpoint inhibitor (ICI) therapy, and was the first histology‐agnostic biomarker used for this purpose [5, 6, 7]. In May of 2017, the PD‐1 inhibitor pembrolizumab received accelerated approval by the United States Food and Drug Administration (FDA) for the treatment of unresectable or metastatic dMMR/MSI‐H solid tumors with disease progression following prior treatment, for which there were no alternative treatment options [8]. This was followed by tumor‐agnostic approvals for patients with advanced or recurrent dMMR/MSI‐H solid tumors with progression after chemotherapy in other regions, including China in 2021 [9].

The proportion of patients with dMMR or MSI‐H varies across different cancer types. dMMR/MSI‐H has an overall prevalence of 10%–15%, and has been established as a key biomarker for response to some treatments for colorectal cancer (CRC) [10]. Other cancer types have been associated with dMMR and MSI‐H, indicating the requirement for further research on the impact on additional solid tumor types [11]. Immunotherapy has been seen to be effective for the treatment of dMMR solid tumors, including endometrial and colorectal cancers [12, 13]. Integrating immunotherapy/PD‐1 inhibitors into treatment protocols for patients exhibiting dMMR may improve patient outcomes and addresses the requirement for novel treatment options within China.

To date, there is limited population‐specific data on the prevalence of dMMR in advanced solid tumors in China [14]. Due to the limited data and the challenges posed by varying diagnostic and treatment patterns, investigation of the prevalence of dMMR across various tumor types in China is of high importance. By gathering data from patients within China, we report the dMMR prevalence and current treatment landscape of advanced dMMR solid tumors.

## 2 Methods

### 2.1 Study Design

This observational study aimed to determine the prevalence of dMMR in patients with metastatic or unresectable solid tumors in patients with endometrial, ovarian, cervical, biliary tract, and gastric cancers in China. Tissue samples were collected to assess the dMMR status of patients from five institutions in China. The total cohort of patients included in this study was enrolled between May 2020 and December 2021, including prospective testing, and clinical data collection. The list of participating institutions can be found in Supplemental Material A. All patients provided informed consent prior to enrolment in the study as required and all procedures were in compliance with each institution's IRB (No. S‐K1077, 2020PS698K/2020PS698K(X1), B2020‐251‐01, 2020‐058‐01 and B2020‐230R).

### 2.2 Study Population

Eligible patients with a histologically or cytologically documented advanced solid tumor of interest (endometrial, ovarian, cervical, biliary tract and gastric cancer [including gastroesophageal junction]) were identified through hospital pathology and medical oncology departments from the five centers. Patients were ≥18 years of age at diagnosis, either treatment‐naïve or previously treated with a tumor tissue sample that was no more than 3 years old.

### 2.3 Tissue Sample Collection and Analysis

A total of 9 slides with 4–5 µm freshly cut formalin‐fixed paraffin‐embedded (FFPE) sections from surgical resections or core needle biopsies were recommended for each patient. One slide was hematoxylin and eosin (H&E) stained, four were single‐stained with dMMR markers (MLH1, PMS2, MSH2, and MSH6), and four were stained with negative control antibodies using the VENTANA MMR RxDx panel according to the manufacturer's protocol. Pathological evaluations and dMMR assessment were performed by a hospital pathologist.

### 2.4 Data Collection of Demographic Information

Using the electronic medical record system or chart review for each study center, demographic information, clinicopathological data, and other biomarker data were extracted.

### 2.5 Statistical Analysis

For the primary objective of determining the prevalence of dMMR for each tumor type, data were counted numerically, and proportions calculated with 95% confidence intervals. The dMMR status by demographic and clinicopathological characteristics along with treatment history were analyzed descriptively.

## 3 Results

### 3.1 Patient Characteristics

**FIGURE 1:**
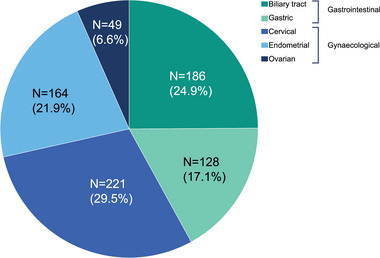
Patient enrolment across cancer types (*N* = 748). *N*, number of patients in the corresponding group.

The full analysis set (FAS) comprised a total of 748 patients, representing five tumor types (gastric, biliary tract, endometrial, ovarian, and cervical cancers). The baseline characteristics of patients in the FAS were generally balanced across the dMMR and MMR proficient (pMMR) populations (Table 1). Of the patients with samples measurable for MMR status, 314 (42.0%) had gastrointestinal and 434 (58.0%) had gynecological tumors (Figure 1). The gastrointestinal tumors consisted of 186 (24.9%) biliary tract and 128 (17.1%) gastric tumor types. Of gynecological tumors, 221 (29.5%) were cervical, 164 (21.9%) endometrial, and 49 (6.6%) ovarian tumors.

**TABLE 1:** Characteristics of patients with all cancer types.

Characteristic	Overall (*N* = 748)	dMMR (*N* = 70)	pMMR (*N* = 678)
Age			
18–65 years	555 (74.2)	62 (88.6)	493 (72.7)
≥65 years	193 (25.8)	8 (11.4)	185 (27.3)
Sex			
Female	571 (76.3)	63 (90.0)	508 (74.9)
Male	177 (23.7)	7 (10.0)	170 (25.1)
ECOG PS at diagnosis			
0	97 (13.0)	8 (11.4)	89 (13.1)
1	90 (12.0)	5 (7.1)	85 (12.5)
≥2	6 (0.8)	1 (1.4)	5 (0.7)
Unknown	555 (74.2)	56 (80.0)	499 (73.6)
TNM stage at diagnosis			
Stage III	609 (81.4)	55 (78.6)	554 (81.7)
Stage IV	138 (18.4)	15 (21.4)	123 (18.1)
Unknown^a^	1 (0.1)	0 (0.0)	1 (0.1)

*Note*: Values are *n* (%).

Abbreviations: ECOG PS, Eastern Cooperative Oncology Group performance status; *N*, number of patients in the corresponding group, dMMR, deficient mismatch repair; pMMR, proficient mismatch repair; TNM, tumor, node, and metastasis staging classification.

^a^ TNM staging was not performed when patients presented with advanced disease.

### 3.2 Prevalence of dMMR

**FIGURE 2:**
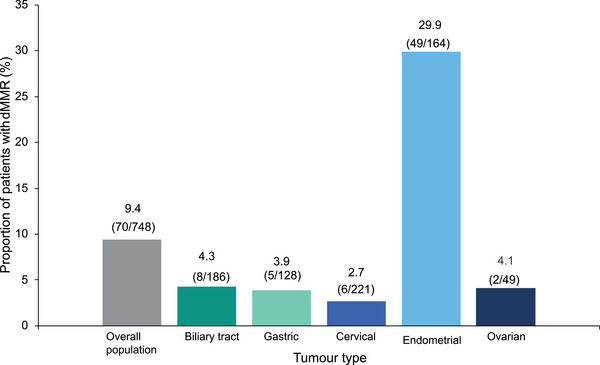
Prevalence of dMMR by tumor type. dMMR, deficient mismatch repair.

The prevalence of dMMR varied across different tumor types, with an overall prevalence of 9.4% (70/748). Of patients with gastrointestinal tumors, the proportion with biliary tract tumors with dMMR was 4.3% (8/186). Patients with gastric tumors had a dMMR prevalence of 3.9% (5/128). Of gynecological tumors, patients with ovarian tumors had a prevalence of dMMR of 4.1% (2/49). Patients with cervical tumors had the lowest prevalence of dMMR of all tumor types with 2.7% (6/221). Patients with endometrial tumors had the highest overall prevalence of dMMR at 29.9% (49/164) (Figure 2).

### 3.3 Demographic and Clinicopathological Characteristics of Patients With dMMR

The prevalence of dMMR was similar across most demographic characteristics. A total of 35.3% (47/133) patients with endometrial tumors who were between 18 and 65 years of age were observed to have dMMR. A total of 6.5% (2/31) patients with dMMR were observed in those ≥65 years of age. All other tumor types were observed to have a comparable prevalence of dMMR, regardless of age (Table 2). Other baseline characteristics had numerically comparable prevalences of dMMR.

**TABLE 2:** Prevalence of dMMR tumors in the overall study population and by tumor type across baseline characteristics.

Characteristic	Overall population (*N* = 748), *n*/*N* (%)	Biliary tract (*N* = 186), *n*/*N* (%)	Gastric (*N* = 128), *n*/*N* (%)	Cervical (*N* = 221), *n*/*N* (%)	Endometrial (*N* = 164), *n*/*N* (%)	Ovarian (*N* = 49), *n*/*N* (%)
Age						
18–65 years	62/555 (11.2)	6/113 (5.3)	1/69 (1.4)	6/200 (3.0)	47/133 (35.3)	2/40 (5.0)
≥65 years	8/193 (4.1)	2/73 (2.7)	4/59 (6.8)	0/21 (0.0)	2/31 (6.5)	0/9 (0.0)
Sex						
Female	63/571 (11.0)	4/85 (4.7)	2/52 (3.8)	6/221 (2.7)	49/164 (29.9)	2/49 (4.1)
Male	7/177 (4.0)	4/101 (4.0)	3/76 (3.9)	NA	NA	NA
Tumor stage at diagnosis						
Stage III	55/609 (9.0)	6/165 (3.6)	3/87 (3.4)	4/192 (2.1)	41/136 (30.1)	1/29 (3.4)
Stage IV	15/138 (10.9)	2/21 (9.5)	2/40 (5.0)	2/29 (6.9)	8/28 (28.6)	1/20 (5.0)
Unknown^a^	0/1 (0.0)	NA	0/1 (0.0)	NA	NA	NA
Tumor grade at diagnosis						
Well differentiated	10/175 (13.1)	1/23 (4.3)	0/2 (0.0)	0/13 (0.0)	9/36 (25.0)	0/1 (0.0)
Moderately differentiated	25/337 (7.4)	6/133 (4.5)	2/42 (4.8)	3/118 (2.5)	14/40 (35.0)	0/4 (0.0)
Poorly differentiated	14/168 (8.3)	1/25 (4.0)	3/76 (3.9)	1/31 (3.2)	9/21 (42.9)	0/15 (0.0)
Unknown	21/168 (12.5)	0/5 (0.0)	0/8 (0.0)	2/59 (3.3)	17/67 (25.4)	2/29 (6.9)

Abbreviations: *N*, number of patients in the corresponding group; *n*, number of patients with dMMR status; NA, not applicable; TNM, tumor, node, metastasis.

^a^ TNM staging was not performed when patients presented with advanced disease.

In the dMMR population across all tumor types, protein loss of MLH1 and PMS2 in 68.6% (48/70) patients was observed most commonly. Protein loss of MSH2 and MSH6 was observed in a total of 18.6% (13/70) patients (Table 3).

**TABLE 3:** Prevalence of MLH1/MSH2/MSH6/PMS2 protein deficiencies in the dMMR population by tumor type in China.

Tumor type	MLH1 and PMS2, *n* (%)	MSH2 and MSH6, *n* (%)	MSH6, *n* (%)	PMS2, *n* (%)
Overall (*N* = 70)	48 (68.6)	13 (18.6)	4 (5.7)	5 (7.1)
Biliary tract (*N* = 8)	5 (62.5)	3 (37.5)	0 (0.0)	0 (0.0)
Gastric (*N* = 5)	4 (80.0)	0 (0.0)	0 (0.0)	1 (20.0)
Cervical (*N* = 6)	4 (66.7)	1 (16.7)	0 (0.0)	1 (16.7)
Endometrial (*N* = 49)	35 (71.4)	9 (18.3)	3 (6.1)	2 (4.1)
Ovarian (*N* = 2)	0 (0.0)	0 (0.0)	1 (50.0)	1 (50.0)

Abbreviations: MLH1, MutL homolog 1; MSH2, MutS homolog 2; MSH6, MutS homolog 6; *N*, number of patients with dMMR status in the corresponding group; PMS2, mismatch repair endonuclease postmeiotic segregation increased 2.

### 3.4 Treatment Pattern at Any Point Since Initial Diagnosis

The proportion of patients with dMMR who had previously received ICIs was 11.4% (8/70), while a total of 4.6% (31/678) patients with pMMR had previously received ICIs. The proportion of patients with dMMR amongst patients who had previously undergone chemotherapy treatment was 70.0% (49/70). A total of 40.0% (28/70) patients with dMMR had previously received radiation treatment. The proportion of patients with pMMR in patients who had chemotherapy totaled 329/678 (48.5%). The proportion of patients with pMMR who had previously undergone radiation therapy was 156/678 (23.0%). All other treatments were generally comparable in terms of the prevalence of dMMR (Table 4).

**TABLE 4:** Treatment history by tumor type.

Treatment	Overall (*N* = 748), *n* (%)	dMMR (*N* = 70), *n* (%)	pMMR (*N* = 678), *n* (%)
Surgery	715 (95.6)	64 (91.4)	651 (96.0)
Radiation	184 (24.6)	28 (40.0)	156 (23.0)
Chemotherapy	378 (50.5)	49 (70.0)	329 (48.5)
Targeted therapy	69 (9.2)	6 (8.6)	63 (9.3)
Immune checkpoint inhibitor	39 (5.2)	8 (11.4)	31 (4.6)

Abbreviations: dMMR, deficient mismatch repair; *N*, number of patients in the corresponding group; pMMR, proficient mismatch repair.

## 4 Discussion

This study aimed to investigate the prevalence of dMMR across multiple advanced tumor types in China and is the first to our knowledge to provide real‐world evidence of MMR status in a Chinese population. The overall prevalence of dMMR in stage III and stage IV tumors in this study (9.4%) was seen to be consistent with that of previous studies of advanced tumors in Korea, Japan, Singapore, and the United States across multiple tumor types [11, 15]. The prevalence of dMMR in stage I and II tumors has been seen to be generally higher than in stages III and IV; however, dMMR is still observed in a notable amount of advanced solid tumors [11]. Analysis of international pooled data of the prevalence of dMMR in both early and advanced stages has found substantial variation in the prevalence of dMMR across multiple tumor types [14]. The results of the current study in patients with advanced‐stage disease and global results suggest that the prevalence of dMMR is not solely dependent upon the race of the patient, tumor location, or disease stage, indicating the benefit of widespread dMMR testing as a standard practice.

The prevalence of dMMR in endometrial tumors seen in the current study is somewhat numerically higher than that observed in multiple studies of the prevalence of dMMR in advanced or metastatic endometrial solid tumors [15, 16, 17]. These results are further indication of the importance of timely testing for dMMR as a standard practice, regardless of tumor location and stage. Testing and detection of dMMR is vital to developing an understanding of the population, which may benefit from treatments such as ICIs [6, 7]. In the United States, pembrolizumab received accelerated FDA approval for pan‐tumor treatment of dMMR and MSI‐H in 2017 for patients who have progressed following prior treatment without satisfactory alternative treatment options or for patient with CRC who have progressed following certain types of chemotherapy [8]. Full approval for MSI‐H/dMMR solid tumors across over 30 tumor types occurred in 2023 following key clinical studies [7]. The majority of ICIs in China were subject to conditional approval, while those achieving full approval were for the treatment of non‐small‐cell and small‐cell lung cancer [18]. Approval of pembrolizumab in China for dMMR and MSI‐H occurred more recently, in May 2021 after the commencement of this study. The relatively low proportion of dMMR‐positive patients who had previously received immunotherapy in the present study (11.4%) can be explained by the relative recency of these developments in China. In patients with advanced‐stage cancers and dMMR, ICIs have been investigated and have shown a clinical benefit in patients with endometrial, ovarian, cervical, biliary tract, and gastric cancers [6, 7, 19, 20]. However, additional studies are required to further analyze the efficacy of ICIs in patients with locally advanced or metastatic disease.

There are several methods by which MMR status can be detected. IHC testing carries the advantages of being simple, with a relatively low cost and low requirement for specialized equipment; however, it can yield some false negative results and accuracy of detection can vary based upon the antibody type and gene mutation [21]. Given the greater sensitivity and accuracy of polymerase chain reaction (PCR) detection, this method is recommended for use as a confirmatory test alongside other testing methods to ensure that all possible cases of dMMR are detected [21, 22]. The American Society of Clinical Oncology (ASCO)‐endorsed College of American Pathologists (CAP) clinical practice guideline recommends MMR testing by PCR testing for gastroesophageal and small bowel cancer, and MMR‐IHC for gastroesophageal and small bowel and endometrial cancers who are being considered for ICI therapy [23, 24]. Similarly, the Chinese Society of Clinical Oncology (CSCO) guidelines on gastric cancer recommend testing for the presence of dMMR by MMR‐IHC in order to guide treatment [25]. The present study utilized the Ventana MMR RxDx assay, which has been approved by the FDA as a companion diagnostic [26]. Evaluation of this panel has shown that in comparison with PCR‐based analysis, the Ventana MMR RxDx panel had improved accuracy in detecting MMR status [27]. The present study highlights the importance of testing for dMMR, providing insights into the dMMR status of patients across solid tumor types in a Chinese population utilizing this assay. Following identification of dMMR in patients with gastric tumors, CSCO guidelines recommend neoadjuvant treatments such as immunotherapy as a first‐line treatment [25]. The pan‐Asian guidelines for the diagnosis and treatment of endometrial cancer recommend chemotherapy as first‐line therapy and immunotherapy as second line following confirmation of dMMR status [28]. The recommendations for biomarker‐selected therapies highlight the importance of standardization of dMMR testing for patient identification, improvements to clinical practice, and overall treatment outcomes.

This study has several limitations, including potential variability that may arise in data generation across different study sites. The stage of the tumor may also influence biomarker expression [22]; however, all tumors tested here were stage III or IV, minimizing the impact. One strength of this study was the pre‐specification of the Ventana MMR RxDx Panel for all dMMR biomarker testing across all study sites in China, which ensured a more accurate interpretation of MMR results.

In conclusion, this study provides epidemiological data for MMR status across various tumor types in China. A higher proportion of patients with dMMR tumors received ICIs compared with patients with pMMR tumors. While studies have investigated the prevalence of dMMR/MSI‐H, they have largely focused on a singular tumor type, and to our knowledge, no study of this nature has been performed in China [29, 30, 31, 32]. ICI treatment has become an important therapeutic strategy for patients with dMMR solid tumors. In analyzing the incidence of dMMR, patients could benefit from receipt of ICI therapy in a clinical setting. These data highlight the importance of routine dMMR biomarker testing in patients with advanced solid tumors in China to optimize treatment decisions. Comprehensive analysis of the prevalence of dMMR is essential to further develop treatments and improvements to the standard of care.

## Conflicts of Interest

Yan Wang was formerly an employee of MSD China (at the time of the study); he is currently an employee of Takeda Development Center Asia and owns stock in Takeda. Song Ling Poon, Michael Thomas Wong, and Rubentiran Ramar are employees of Merck Sharp & Dohme LLC, a subsidiary of Merck & Co., Inc., Rahway, NJ, USA, who may own stock and/or hold stock options in Merck & Co., Inc., Rahway, NJ, USA. All other authors have no competing interests to declare.

## Supporting information




**Supplemental Material A**: List of participating institutions.
