# Supplementary material for: Prevalence of DNA Mismatch Repair Deficiencies in Multiple Solid Tumor Types in China
Source: J Evid Based Med. 2025 Nov 28;18(4):e70081. doi: 10.1111/jebm.70081 (PMC12750482; doi:10.1111/jebm.70081)
Supplement: Supplementary file 1 — Supplemental Material A: List of participating institutions. [file JEBM-18-0-s001.docx]

**Supplemental Materials**

**Supplemental Material A. List of participating institutions.**

1. Peking Union Medical College Hospital, Chinese Academy of Medical Sciences, and Peking Union Medical College, Beijing, China

2. Shengjing Hospital of China Medical University, Shenyang, Liaoning, China

3. Sun Yat-sen University Cancer Center, Guangdong, Guangzhou, China

4. The Affiliated Drum Tower Hospital of Nanjing University Medical School, Nanjing, Jiangsu, China

5. Zhongshan Hospital, Fudan University, Shanghai, China
